# Improving Medical Student Surgery Notes Through Near-Peer Targeted Education: A Qualitative Analysis

**DOI:** 10.1016/j.jss.2025.02.035

**Published:** 2025-03-21

**Authors:** Ariana Naaseh, Rachel Kalbfell, Carla Koberna, Kerri A. Ohman, Lindsay M. Kranker, Bethany C. Sacks

**Affiliations:** Department of Surgery, Washington University in St. Louis School of Medicine, St. Louis, Missouri

**Keywords:** Documentation, Medical record, Medical student, Notes, Qualitative, Surgery, Surgery clerkship

## Abstract

**Introduction:**

It is necessary for medical students (MSs) to develop proficiency in medical documentation before residency; however, there is limited education focused on this task. We conducted a qualitative analysis of surgical clerkship note evaluations to create an intervention to improve note writing skills and utilized post-intervention assessments to determine the effect on the quality of clerkship student notes.

**Methods:**

An intervention consisting of an in-person senior MS led lecture during the surgery clerkship orientation focused on Subjective-Objective-Assessment-Plan (SOAP) notes was introduced to MS with limited prior note writing education. Examples of complete and accurate surgical SOAP notes were discussed in-person and shared online. Reports from note assessments were analyzed for four clerkship cycles (2 pre-intervention and 2 post-intervention). Descriptive statistics and Fisher’s exact tests were performed to compare assessment responses pre-intervention and post-intervention. Thematic analysis was performed on open-ended responses focused on areas for improvement from the anonymized evaluations.

**Results:**

One hundred seventy-one total note assessments were evaluated (85 pre-intervention and 86 post-intervention). Pre-intervention, MS struggled with note organization and lack of inclusion of patient-centered language within their documentation. Post-intervention, students struggled less frequently with flow and chronology of notes but had continued opportunities to improve in concise and organized presentation of information. Students improved in their ability to provide a one-liner, prioritize a differential diagnosis, and organize and structure their notes effectively. Feedback focused on advanced skills including plan specificity, discharge details and parameters, or advanced physical exam finding documentation.

**Conclusions:**

MS improved their SOAP format note writing skills in response to a targeted near-peer led educational intervention. Remaining growth opportunities in note writing skills appear more connected to evolving clinical reasoning and surgery-specific knowledge which can evolve with ongoing exposure and further education.

## Introduction

Written electronic health record (EHR) documentation is a cornerstone of health-care communication that medical students (MSs) must be proficient in before matriculation to residency. The Liaison on Medical Education, the Association of American Medical Colleges, and Accreditation Council for Graduate Medical Education all recognize written communication and medical record documentation as necessary components of medical and graduate education.^1-3^ In 2012, the Alliance for Clinical Education recommended that medical schools develop a clear set of guidelines related to MS documentation in the EHR to best prepare students for clinical practice.^4^ Despite this, two national surveys performed by the Alliance for Clinical Education and Liaison on Medical Education prior to 2016 have demonstrated that two-thirds or less of students documented notes on their clerkships.^5,6^

Much of this limited use of MS documentation has been partially attributed to Centers of Medicare and Medicaid Services (CMS) restrictions related to using MS notes in billing.^7^ However, in 2018 CMS revised their policy to allow teaching physicians to use MS notes for billable services when under appropriate supervision.^8^ With this policy change, MS documentation is now integrated into the medical record and therefore can be viewable by patients.^9^ While initially there were concerns that expanding documentation to MSs would burden early-career trainees, multiple published studies have demonstrated that including students in documentation can promote positive attitudes toward documentation and enhance understanding of their patients.^10-12^ Considering these changing requirements and new display of MS documentation, the focus has shifted to ensuring that MS notes are of high quality.

Within the existing literature, there is minimal published data on both the existing quality of MS documentation along with interventions and methods to educate students on note-writing or improve their existing skills.^13,14^ Certainly, there is no standard for how to approach educating or providing feedback on note-writing across medical schools. We conducted a qualitative analysis of surgical clerkship MS note evaluations to create an in-person, senior MS led educational intervention aimed to improve surgery clerkship note writing skills. Furthermore, we evaluated postintervention assessments to both determine the effect of the intervention on the quality of MS notes and refine continued education.

## Methods

This study was deemed exempt by the Washington University in St. Louis Institutional Review Board as all data was collected for educational purposes and was later retrospectively and anonymously analyzed.

### Participants and recruitment

All MSs completing their surgery clerkship at the Washington University in St. Louis School of Medicine (WUSM) from January 2, 2024, to September 1, 2024, completed two inpatient or outpatient notes as part of their clerkship requirements. The requirement to submit clinical notes on the clerkships began at the start of the clerkship academic year on January 2, 2024, and minimal formalized instruction was provided to the students across the medical school or within their prior clerkship structures.

To submit a note for assessment, students routed it to a shared grading pool within the EHR to which only assessors and clerkship directors had access. Grading rubrics generated by WUSM were completed in an online WUSM software utilized for all clerkship assessment purposes. Students were introduced to the assessment tool before its implementation and received in-person guidance on the grading criteria for each question. Students submitted notes before the end of their clerkship time that were then graded upon clerkship completion by one of six resident physicians in their professional development time who received online instruction on both the assessment tool and how to complete the assessments. Assessors were aware of which student completed the note and whether they had received the intervention at the time of grading. The resident assessment graders were compensated for their time grading. Students did not receive feedback on their notes until after their surgery clerkship was completed, so were unable to act on feedback during their current clerkship but could do so for future clerkships.

### Assessment instrument

Each assessment form included three rating questions, four binary questions, and one open-ended question. The rating questions asked assessors to select whether students had not performed, partially performed, or completely performed the following: “provides a concise and accurate one-line summary statement or problem representation,” “provides and supports a prioritized diagnostic/management plan or next steps,” and “provides and supports a prioritized differential including ‘no-miss’ or ‘life-threatening’ diagnosis when relevant.” The four binary questions asked assessors to select “yes,” “no,” or “does not apply” to the following statements: “Note was appropriately organized,” “Note was of appropriate length to communicate the necessary information,” “Student incorporated patient preferences and/or values into the diagnostic and/or management plans,” and “Student incorporated hospital system features or limitations into the diagnostic and/or management plans.” The open-ended assessment question asked assessors to “Provide a specific example of an area of targeted improvement or next steps for the student.”

### Data analysis

After all the notes from each clerkship were assessed, an anonymized report was extracted from the grading system and shared with the study team. Every assessment response was coded and scored, amounting to two assessments per student.

Free response answers within the assessment tool were analyzed by two independent reviewers (AN and RK). A codebook was developed through inductive analysis of the assessment responses to the question, “Provide a specific example of an area of targeted improvement or next steps for the student.” without an a priori code list. The codebook was developed after consensus with multiple team members after reviewing the first clerkship assessment responses. After finalizing the codebook, two independent reviewers (AN and RK) scored each assessment response for presence or absence of a given code. The finalized codebook is displayed in Table 1. Themes were developed by the study team and iteratively refined until group consensus was achieved. Regular study team meetings were scheduled between cross-coders and the overall study team to resolve possible discrepancies in coding and thematic analysis. If needed, an additional member of the study team adjudicated decisions in discrepant cases. Each assessment response could only include one instance of each theme but could include multiple codes.

For the first two clerkship cycles of the year (January/February 2024 and March/April 2024), two independent coders (AN and RK) analyzed assessment responses to discern areas for potential improvement and intervention among the surgery clerkship student notes given that the students had minimal prior formalized education on the topic. Themes and codes from the pre-intervention assessment informed our intervention creation, while themes and codes from the post-intervention assessment (May/June 2024 and July/August 2024) were utilized to characterize the effect of the intervention on MS notes.

### Intervention description

After careful review of opportunities for improvement and education from MS note assessment for the first two clerkships of the year, a three-pronged intervention was developed by the research team. The first component was an in-person senior MS led lecture during the MS clerkship orientation week focused on Subjective-Objective-Assessment-Plan (SOAP) notes. This includes key information on the SOAP format, the use of EHR templates, the different contexts in which notes are written and the audience they are written for, and key tips for success. This was introduced during the third clerkship of the year in April 2024. The second component of the intervention included sharing complete and accurate inpatient and outpatient SOAP note examples from surgical subspecialties the students rotate on with the MSs both online and in-person (Supplemental Appendix A). The third component involved in-person with available online reference material on how to use and access EHR templates.

### Statistical analysis

Descriptive statistics were performed on the results of the rating and binary questions within the assessment tool. Rating question responses were analyzed as categorical variables with responses categorized into “completed” (completely performed) and “not completed” (partially performed or not performed). Fisher’s exact tests were used to compare rating and binary assessment responses pre-intervention and post-intervention. *P* values <0.05 were predetermined to be statistically significant. All statistical analyses were completed using R Studio (version 4.2.3; The R Foundation for Statistical Computing, Vienna, Austria).

## Results

### Assessment responses

A total of 171 MS note assessment responses were evaluated, including 85 notes in the pre-intervention period and 86 in the post-intervention period. A summary of the results of the rating and binary questions pre-intervention and post-intervention are listed in Table 2. Improvement was seen in most rating and binary questions from the pre-intervention to the post-intervention period, except for students’ ability to provide and support a prioritized diagnostic/management plan or next steps (97.6% pre-intervention *versus* 96.5% post-intervention, *P* = 1.0). There was a statistically significant improvement in students’ ability to provide and support a prioritized differential (91.8% pre-intervention *versus* 100.0% post-intervention, *P* = 0.01).

### Opportunities for improvement

Thematic analysis of open-ended assessment responses focused on what students could target for improvement across all four clerkships demonstrated seven distinct themes (Table 3). In the pre-intervention period, MSs struggled with note organization, omission of key aspects of the SOAP note format, and utilizing concise and patient-centered language. In the post-intervention period, students struggled less frequently with organization, flow, and chronology of notes, but demonstrated continued opportunities to improve in concise presentation of information along with their documentation of advanced clinical reasoning.

#### Theme 1: challenges with organizing and constructing plans

MSs often utilized premade EHR templates created for billing purposes to construct their notes and therefore their plans. Many noncritically ill patient notes were organized by system, whereas they would likely be easier to digest if shared in a problem-based format. One of the most common suggestions from assessors was to “organize your plan in some way”, with suggestions often including example problem-based hashtags such as “#postoperative pain” or “#acute blood loss anemia” to illustrate for students how best to organize the information in their plan. Students struggled with the balance of quantity of information to include in their plans. Sometimes when writing follow-up notes for established patients, students had trouble discerning how to document that problems were resolved or no longer relevant without lengthening the note.

#### Theme 2: difficulty with correct placement of information in the SOAP format and note formatting

In writing SOAP notes, students struggled with placing the subjective, objective, assessment, and plan in the correct areas of the note. Frequently misplaced note components included placing objective information into the subjective section, including the assessment within the history of present illness, or adding in clinical reasoning throughout the subjective and objective components. For example, a student assessed the patient as “appearing fluid overloaded” within the physical exam documentation rather than the objective finding of “2+ pitting edema in the lower extremities.” Chief complaints were not frequently documented “in the patient’s words.” Many students utilized abbreviations in their notes and were discouraged against using them, especially those that are not well-established or known by their assessors.

#### Theme 3: inaccuracies or inconsistencies in documented information

Some MS note documentation contained inaccurate or discrepant information from other aspects of the note. Assessors ensured that they pointed out when templates were used but not tailored to this specific presenting patient or old versions of an exam or note were carried forward that were in fact no longer accurate. The need for note information to be accurate was highlighted in the context of utilizing notes as a form of communication with consulting providers or other team members and in the dangers of perpetuating inaccurate patient information in the medical record.

#### Theme 4: excluded or incomplete aspects of documentation

Although MSs adhered to the SOAP format, there were often aspects of the notes that were omitted or incomplete. In the pre-intervention period, many students did not include one-liners, chief complaints, or assessment statements. Throughout the entirety of the assessment period, students struggled with including a complete review of systems and representing all appropriate systems in the physical exam. In terms of physical exam documentation, students ranged from documenting only systems affected by the specific surgery performed and omitting systems such as cardiovascular and respiratory or omitting key surgical details such as incision character or drain location and output.

#### Theme 5: trouble balancing succinct and concise documentation with inclusion of all relevant information

When students included one-liners and assessments, these generally were not succinct or formatted in such a way to be clear and informative to the reader. For example, many students repeated their one-liner as their assessment and included information such as all past medical history rather than their impression of the patient in their presenting stage. One-liners from student notes were often “very long and cluttered” especially considering patient presentations in highly specialized clinics where visits were often focused on a key body system or chief complaint. On the other hand, some students favored brevity and sacrificed inclusion of key details such as date of the procedure or any complications relevant to their presentation.

Students struggled with distilling complex patient histories. Students usually included all details of their conversations with patients even when some aspects were not relevant to the chief complaint to ensure they were documenting the patients’ subjective experience completely. Rarely did students demonstrate utilization of clinical reasoning to only include what was relevant to the patient’s presentation in outpatient notes, but students often did so within the interval history for inpatient postoperative patients where it seemed as if they had a more straightforward structure and understanding for what was relevant for these patients.

#### Theme 6: minimal inclusion of patient-centered language and perspectives

Many students did not include patient-centered language or perspectives in their documentation. This was most evident in how patients documented subjective histories for critically ill or pediatric patients but was also evident in the conceptualization of their plans and noticeable lack of discussion of patient preferences especially in clinical scenarios where multiple options were presented for the patient.

#### Theme 7: performing and documenting advanced clinical reasoning

Especially in the postintervention period, most of the feedback focused on more advanced documentation and clinical reasoning skills including plan specificity, coordination of multidisciplinary care, discharge details and parameters, or advanced physical exam finding documentation. Many of the assessors challenged the students to include more documentation of how they came to a specific clinical diagnosis and why the patient’s plan was formulated as it was both for the readers understanding and to ensure the students were learning through the patient encounters that they documented.

## Discussion

In this new era of medical documentation, the importance of assessment of MS note documentation along with education on high-quality EHR communication cannot be understated. Our single-institution study evaluated areas for improvement in MS documentation and utilized these deficiencies to design a multicomponent educational intervention to address these gaps in the learner’s knowledge. Our findings are similar to those previously published in the literature that demonstrate that without formalized education MS notes can be incomplete or inaccurate.^15^ Our intervention addressed structural issues with note organization and stressed the importance of utilizing concise and patient-centered language when documenting surgical patient encounters in the inpatient and outpatient setting. Even after students were educated on SOAP notes, continued deficiencies were observed in documentation of clinical reasoning which has previously been demonstrated even in more senior MSs.^16,17^ Our findings can likely be attributed to both student unfamiliarity with note-writing, especially preintervention, and deficits in student surgical knowledge required for accurate and complete documentation.

The low quality of existing MS notes can be attributed to lack of formal instruction, among other possible impacting factors.^18^ To date, minimal educational interventions have been published to improve MS documentation since the 2018 CMS changes. A near-peer targeted feedback system for MS notes was implemented with success in improving note quality and demonstrated long-term positive effects.^19^ In 2023, students on the pediatric clerkship demonstrated decreased note length and improved note quality after participation in a didactic session on note-writing and templates used in the EMR.^20^ Our study and the existing literature demonstrate that when implemented, targeted educational interventions to improve student note-writing skills are successful.

It can be challenging for medical educators to structure an intervention to improve student note-writing skills when no best practice consensus or guidelines exist. It has been demonstrated that near-peer learning is effective both within and outside of the surgery clerkship context.^21-24^ Benefits to a near-peer teaching model can include providing students with a positive and supportive learning environment and providing senior MSs the opportunity to be educators.^25,26^ In addition, multi-prong educational interventions in the medical setting are feasible and successful.^27^ Our intervention combined evidence-based methods of near-peer and multi-prong teaching approaches to optimize student learning.

The most optimal ways to assess and provide feedback on core MS skills such as medical documentation is an area of constant investigation and innovation. There is limited research available specifically on providing feedback to MSs, who are so new to medical notes, on their documentation. A recent scoping review on the process of providing feedback on learners’ note-writing abilities demonstrated that feedback was beneficial, direct written comments and evaluation tools were the most common feedback methods, feedback on notes in real clinical settings were limited, and that iterative feedback was most beneficial for learners.^28^ We know from other areas of investigation within medical education, that the most important feedback is clear, specific, timely, and actionable.^29^ A limitation of the current note feedback model is that students do not receive formal feedback until they are finished with their clerkship, which prevents them from utilizing the feedback to improve on the second SOAP note they are assessed on.

Feedback quality can be highly variable and the distinction between our quantitative and qualitative assessment results demonstrates this. Had we solely based our need for further investigation on the quantitative rating and binary questions, there seemed to be minimal need to educate students on high-quality note writing given the overall high scores. The current assessment rubric standards and rating scales perhaps are not helpful and appropriate in improving MS documentation given the lack of actionable and constructive feedback they provide. The Physician Documentation Quality Instrument-9 has been previously validated and used to assess both the quality of physician and now MS notes.^30,31^ Medical schools can explore using this tool which focuses on nine attributes of documentation: up-to-date, accurate, thorough, useful, organized, comprehensible, succinct, synthesized, and internally consistent.^32^ Using objective scoring metrics can also assist in reducing bias in MS grading and feedback, especially when utilizing global rating scales rather than binary checklists.^33-35^

Beyond designing an intervention and understanding best practices for feedback delivery, there are notable barriers and challenges to implementation which can include lack of available assessors and the time required to provide personalized feedback to students.^36,37^ Furthermore, it can be challenging to integrate an intervention within an already demanding clerkship schedule and to decide what the appropriate balance is between synchronous and asynchronous learning to optimize student education.^38,39^ Our intervention was easily integrated into the existing clerkship structure within an already scheduled near-peer lecture and provided materials that students could asynchronously access for the entirety of their clerkship and beyond. To overcome lack of available and engaged assessors, we utilized residents in their professional development time with no current clinical duties to provide actionable and appropriately timed feedback. While this may not be possible in every environment, our model also compensated assessors for the time spent grading which can assist in alleviating the administrative burden that providing feedback can incur on more senior trainees and faculty.

Although this is a single institution study which could limit the generalizability of findings, given the lack of widely available data on such an important topic we believe our findings will be of interest to medical and surgical educators. Since assessors were not blinded to the intervention launch, it is possible that their evaluations of postintervention notes were biased by their awareness that students had received the intervention. Due to the new clerkship requirements at the start of our study period, we had the unique opportunity to educate students who had limited prior formalized knowledge on medical documentation, allowing us to assess the effect of our intervention on subsequent student performances. However, we likely cannot attribute all the students’ improvement to solely our curriculum as in later clerkship cycles students had more exposure to note writing with increased repetition and students could have been exposed to some informal education through other clerkships. Even with possible education on other clerkships, our intervention clearly focuses on documentation for surgery patients in the inpatient and outpatient setting, which would likely not be emphasized elsewhere in their clerkship education. Furthermore, our study still highlights that MSs had important areas for improvement in documentation even as they progressed through their clerkships and had more exposure to note writing.

### Conclusions

MS documentation should be a major focus for medical and surgical educators given the increased transparency of the medical record to patients, possibility for utilizing MS notes for billing purposes, and students’ progression to residency where medical documentation is an integral part of their future role. Our study identified key areas in which students struggle to document patient encounters and presented a multipronged intervention aimed to improve MS note-writing education. Improving MS documentation skills on the surgery clerkship likely will require targeted training in note-writing techniques and foundational surgical knowledge. Future study is needed to assess how MSs experienced the intervention and how they would prefer to receive note-writing education and feedback. Furthermore, continued evaluation of MS notes is necessary as it can inform future intervention creation and redesign at our institution and nationally.

## Supplementary Material

1

Supplementary data related to this article can be found at https://doi.org/10.1016/j.jss.2025.02.035.

## Figures and Tables

**Table 1 –:** Codes and definitions utilized for thematic analysis.

Code	Definition
Organization	Flow of the note, chronological, readability of history and plan
Notes formatting	Adheres to SOAP format, includes correct information in correct section
Section issues	Issues specific to one section of the note such as the physical exam, plan, one-liner – can include omission of key information or omission of a section
Surgery-specific communication	Concise and focused language, tailored to surgical concerns
Relevance of information	Inclusion of key and relevant details (e.g. – postoperative day, operation, pertinent positives/negatives), focused physical exam with incisions, drains, lines, etc.
Patient experience	Inclusion of patient’s point of view, emotions, thoughts, perspectives

**Table 2 –:** Summary of quantitative assessment questions, preintervention, and postintervention.

Three rating questions	Preintervention (*n* = 85)		Postintervention (*n* = 86)		*P* value
	Partially performed or needed attention	Completely performed	Partially performed or needed attention	Completely performed	
Provides a concise and accurate one-line summary statement or problem representation	7.1%	92.9%	1.2%	98.8%	0.06
Provides and supports a prioritized diagnostic/management plan or next steps	2.4%	97.6%	3.5%	96.5%	1.00
Provides and supports a prioritized differential including ‘no-miss’ or ‘life-threatening’ diagnosis when relevant	8.2%	91.8%	-	100.0%	0.01
Four binary questions	No	Yes	No	Yes	
Note was appropriately organized	2.4%	97.6%	-	100.0%	0.25
Note was of appropriate length to communicate the necessary information	2.4%	97.6%	1.2%	98.8%	0.62
Student incorporated patient preferences and/or values into the diagnostic and/or management plans*	20.6% (*n* = 7)	79.4% (*n* = 27)	-	100.0% (*n* = 7)	0.32
Student incorporated hospital system features or limitations into the diagnostic and/or management plans*	33.3% (*n* = 2)	66.7% (*n* = 4)	-	100.0% (*n* = 4)	0.47

* Item only scored when applicable to note content.

**Table 3 –:** Themes and representative assessment quotes.

Theme	Representative assessment quotes
Challenges with organizing and constructing plans	- “Would be more specific with plan. Some of what you wrote does not provide much information while lengthening the note.”
	- “Would consider breaking down postoperative problems in the plan with separate hashtags instead of one large #postoperative problem – or can do by systems.”
	- “Your plan should be organized in some way. Chief complaints have been resolved is insufficient and generic.”
Difficulty with correct placement of information in the SOAP format and note formatting	- “Try not to mix in objective data with your subjective/interval history/HPI.”
	- “Your chief complaint should be in the patient’s words. Save your diagnosis for your assessment and plan!”
	- “Try not to use abbreviations especially those that are not commonly used or well-known.”
	- “For physical exam, would provide more details than ‘appears well-perfused’ under Cardiac–unclear what this means and for exam, would be better to instead of providing interpretation, give actual physical exam findings (e.g., palpable DP pulses bilaterally)”
Inaccuracies or inconsistencies in documented information	- “Your physical exam says normal shaped breasts, but you are seeing a patient who is surgically s/p bilateral mastectomy. Ensure your exam is accurate and up to date.”
	- “Be sure to double check the dates that you are using – the dates of the patients transplant are not the same within your assessment as they are within your HPI”
Excluded or incomplete aspects of documentation	- “Would push you to write out a full ROS for practice. You shouldn’t default to writing per HPI unless you actually ask about 10 different systems within the HPI.”
	- “Add a one liner at the top of your note to frame who this patient is.”
	- “Expand on your physical exam to include more than just three systems.”
Trouble balancing succinct and concise documentation with inclusion of all relevant information	- “Could be more concise in both interval history and assessment. Do not need to include the full PMH list in your assessment.”
	- “The one liner is very long and cluttered. Consider cleaning it up to be a little easier to read and follow especially for a specialty oncology clinic.”
	- “Would recommend adding in some key details within your one-liner like date of procedure and any complications/issues thereafter in such a complex surgical patient.”
	- “Make your one liner more concise and ensure it has all of the details I need to understand who the patient is - postoperative visit for what?”
Minimal inclusion of patient-centered language and perspectives	- “Listing out the events over the last 24 h is helpful but you have not included any subjective experience from the patient in your HPI.”
	- “Try to include some more of the patient’s perspective of their liver disease and why being here and getting a consultation for a transplant is important to them!”
	- “You did a great job of detailing some of the patient’s social history including her move and her lapse in medication during that time in which she moved. This is a great opportunity to document why - did the patient have trouble establishing care in a new country? Was she adjusting to the move? Was she establishing insurance coverage?”
	- “Could include more of the patient’s voice in the subjective. Does the patient come in with any preferences? You included that in the A/P but it should be moved up to the subjective!”
Performing and documenting advanced clinical reasoning	- “Could provide details regarding discharge planning if relevant in the short-term.”
	- “When planning to advance diet, typically can advance pain regimen to oral from IV – take note of those details and adjust them in your plan.”
	- “Try to include your detail in your plan about why the patient does not require follow-up and/or is cleared from being seen back by surgery.”
	- “Specifically for an umbilical hernia, you could add the size of the defect if palpable or a picture of the defect for more detail for your exam.”
	- “Would recommend fleshing out your assessment a bit by adding a differential diagnosis and why you think this could be either appendicitis *versus* hernia *versus* bowel obstruction.”
