# Supplementary material for: Improving Medical Student Surgery Notes Through Near-Peer Targeted Education: A Qualitative Analysis
Source: J Surg Res. Author manuscript; Available in PMC 2026 Apr 27. (PMC13112468; doi:10.1016/j.jss.2025.02.035)
Supplement: 1 [file NIHMS2160578-supplement-1.pdf]

Supplemental Appendix A: Sample Outpatient Subjective, Objective, Assessment, and Plan Note provided to medical students.

Colon & Rectal Surgery Clinic New Patient

CC: Chronic Anal Fissure

HPI: 75 y.o. M/F with history of chronic constipation, internal/external hemorrhoid, and chronic anal fissure who presents today to discuss further management recommendations for their anal fissure. The patient was initially diagnosed with an anal fissure four years ago and saw another colorectal surgeon at that time who opted to manage with topical nitroglycerin. About one year ago, they had a recurrence of their symptoms including 10/10 pain during and after defecating. This pain was partially relieved by topical nitroglycerin and hot showers. Now, they report that they occasionally have burning 2-3/10 pain while defecating, primarily when they are not able to completely empty their bowels. They are no longer using the topical nitroglycerin now that their symptoms are more controlled but is very concerned about recurrence.

Notably, they previously had years of chronic constipation. The patient underwent EGD and colonoscopy to determine the etiology of the constipation about 1 year ago and was diagnosed with segmental colitis and diverticulosis. At that time, the patient was started on mesalamine and reports and remarkable improvement in their stool consistency. They deny recent bloating, abdominal pain, or melena. However, the patient notes occasional bright red blood per rectum with irritation of their hemorrhoids.

Past Medical History:
Segmental colitis & diverticulosis
Chronic constipation
Internal & external hemorrhoids
HTN
HLD

Past Surgical History:
None

Medications:
Reviewed in chart

Allergies:
Ciprofloxacin – reaction: hives

Family History:
Noncontributory. No family history of colorectal cancer or inflammatory bowel disease.

Social History:
The patient reports they are a current smoker, about ½ pack per day. Denies alcohol or illicit drug use. Currently is employed doing manual labor and lives at home with their family.

Review of Systems:
Constitutional: No fever, no chills, no unintended weight loss
Neurological: No headaches, no dizziness, no vision changes
Cardiovascular: No chest pain, no palpitations
Respiratory: No shortness of breath, no coughing
Psychiatric: No mood changes, no depression
Lymphatic: No swollen lymph nodes

Hematology: No easy bruising/bleeding
Integument: No itching, no non-healing sores
Oral: No mouth sores
Musculoskeletal: No joint stiffness
Gastrointestinal: No nausea, no vomiting, no diarrhea, **+constipation, +hematochezia, +pain with defecation**
Genitourinary: No hematuria

Physical exam:
Vitals

BP	140/80
Pulse	91
Temp	36.8 °C (98.3 °F)
SpO2	97%
BMI	29.46 kg/m²

General: Well developed, well nourished
Psych: Alert and calm
HEENT: normocephalic/atraumatic, anicteric
Pulm: nonlabored breathing on room air, lungs clear to auscultation bilaterally.
CV: well-perfused, regular rate, normal rhythm, no murmur auscultated
GI: abdomen nondistended, nontender, no masses, no hepatomegaly.
Anorectal: Multiple hemorrhoids and skin tags visualized on external exam. Healing fistula with scant discharge visualized in the 12 o'clock position. DRE with increased tone, no stricture, mass, abscess
Neuro: grossly non-focal, ambulatory, moving all extremities
Skin: warm and dry
Musculoskeletal: no gross deformities

A medical assistant chaperone was present for the exam.

Assessment/Plan:
Mr./Ms. *** is a 75 yo M/F with a healing anal fissure whose symptoms have been improving with medical management. At this point, surgical management would not be recommended. Furthermore, the chronic constipation that was likely the cause of the anal fissure has been resolved with treatment. Hopefully, this will result in less aggravation of the fissure.

Together we discussed continued medical management such as installing a bidet with warmed water and taking warm showers for tone improvement. We also recommended using topical nifedipine or nitroglycerin. We discussed the risks and benefits of a lateral internal sphincterectomy if the anal fissure were to worsen. Risks include infection, bleeding, and small risk of fecal incontinence. We conveyed that risks of surgery can be avoided with medical management especially because her symptoms are improving.

#Anal fissure
- recommend bidet with warmed water
- topical nifedipine or continue topical nitroglycerin PRN
- topical lidocaine jelly PRN

#Hemorrhoids
- bidet will help reduce irritation with wiping

#Chronic constipation
- resolved
